# A Covalent Chemical Probe for *Chikungunya* nsP2 Cysteine Protease with Antialphaviral Activity and Proteome-wide Selectivity

**DOI:** 10.21203/rs.3.rs-5363451/v1

**Published:** 2024-11-12

**Authors:** Anirban Ghoshal, Edwin G. Tse, Mohammad Anwar Hossain, Kesatebrhan Haile Asressu, Eric M. Merten, John D. Sears, Stefanie Howell, Sumera Perveen, Jane Burdick, Noah L. Morales, Sabian A. Martinez, Isabella Law, Bennett J. Davenport, Thomas E. Morrison, Zachary J. Streblow, Daniel N. Streblow, Angie L. Mordant, Thomas S. Webb, Aurora Cabrera, Laura E. Herring, Cheryl H. Arrowsmith, Kenneth H. Pearce, Nathaniel J. Moorman, Mark T. Heise, Rafael Miguez Couñago, Peter J. Brown, Timothy M. Willson

**Affiliations:** † Structural Genomics Consortium, UNC Eshelman School of Pharmacy, University of North Carolina at Chapel Hill, Chapel Hill, NC 27599, USA.; # READDI AViDD Center, University of North Carolina at Chapel Hill, Chapel Hill, NC 27599, USA.; ‡ UNC Eshelman School of Pharmacy, Center for Integrative Chemical Biology and Drug Discovery, University of North Carolina at Chapel Hill, Chapel Hill, NC 27599, USA; § Department of Microbiology and Immunology, University of North Carolina at Chapel Hill, Chapel Hill, NC, 27599, USA; ¥ Structural Genomics Consortium, University of Toronto, Toronto, Ontario, M5G 1L7, Canada; ⊥ Department of Genetics, University of North Carolina at Chapel Hill, Chapel Hill, NC, 27599, USA; ° Department of Immunology and Microbiology, University of Colorado School of Medicine, Aurora, CO 80045, USA.; ∥ Vaccine & Gene Therapy Institute, Oregon Health & Science University, Beaverton, OR, 97006, USA; ¶ UNC Metabolomics and Proteomics Core, University of North Carolina at Chapel Hill, Chapel Hill, NC, 27599, USA; ∇ Center of Medicinal Chemistry, Center for Molecular Biology and Genetic Engineering, University of Campinas, 13083-886-Campinas, SP, Brazil

## Abstract

RA-0003022 (**3**) was identified as a high-quality covalent chemical probe for *Chikungunya* nsP2 cysteine protease (nsP2pro). Isoxazole **3** covalently captured the active site C478 and inactivated the enzyme with a *k*_inact_/*K*_i_ ratio of 6000 M^–1^s^–1^. A negative control analog RA-0025453 (**4**) retained the covalent warhead but demonstrated >100-fold decrease in enzyme inhibition. Isoxazoles **3** and **4** were stable across a wide range of pH in solution and upon prolonged storage as solids. The covalent chemical probe **3** was inactive across a panel of 23 human and viral cysteine proteases and demonstrated remarkable proteome-wide selectivity by two chemoproteomic methods. Isoxazole **3** reduced viral titer against infectious isolates of *Chikungunya*, *Mayaro*, and Venezuelan Equine Encephalitis viruses demonstrating its activity on both New and Old World alphaviruses. Isoxazole **3** and its negative control **4** will find utility as covalent chemical probes to study the role of the nsP2pro in alphaviral replication and virulence.

## Graphical Abstract

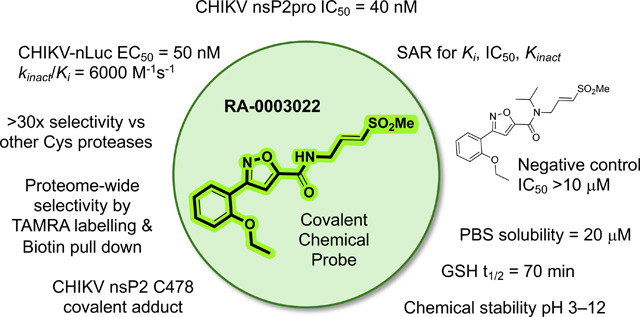

## 1. Introduction

Alphaviruses, a genus of arthropod-borne viruses, continue to pose significant threats to human health, particularly in regions where their vectors are prevalent.^1, 2^ *Chikungunya* virus (CHIKV), which causes chronic arthritis, has infected millions of individuals in the Americas, Africa, and Asia.^3^ Recent outbreaks in New England of Eastern Equine Encephalitis virus (EEEV), whose symptoms can often be fatal or lead to long-term neurological damage, have led to multiple public health warnings and heightened surveillance efforts.^4^ Other alphaviruses of concern include Venezuelan Equine Encephalitis virus (VEEV) and Mayaro virus (MAYV). The endemic global CHIKV infections and localized EEEV outbreaks not only underscore the persistent risk of alphavirus infections but also highlight the urgent need for effective therapeutic interventions, as there are currently no approved antiviral drugs specifically targeting these viruses.^5^ Among the most promising therapeutic targets is the non-structural protein 2 protease (nsP2pro), an alphaviral cysteine protease that plays a crucial role in the viral life cycle.^6^

Cysteine proteases are often targeted using covalent inhibitors. Although inhibitors that target the catalytic cysteine show potent inhibition, a challenge remains to achieve selectivity against the remaining proteome to minimize off-target effects. In their recent review, Hartung et al.^7^ outlined several key criteria for high-quality covalent chemical probes, emphasizing the need for specificity in target engagement. A primary requirement, in addition to potency, selectivity, and the absence of moieties that could lead to promiscuity or assay interference, is that the chemical probe should have a well-defined site of covalent interaction, labeling the primary target without affecting other proteins.

**Figure 1.**
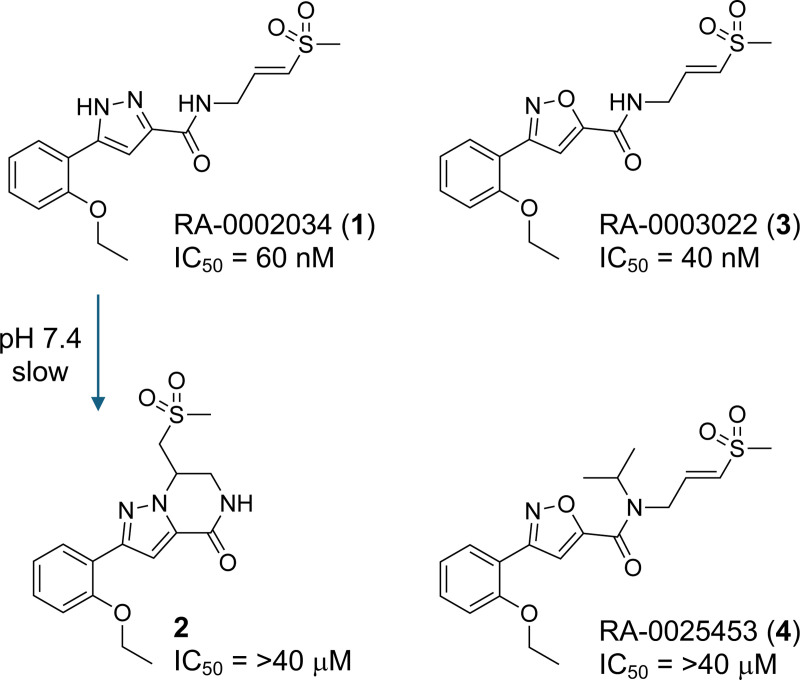
nsP2pro inhibitor RA-0002034 (**1**) undergoes slow intramolecular cyclization to the inactive **2**. Isoxazole RA-0003022 (**3**) is chemically stable and a potent nsP2pro inhibitor. RA-0025453 (**4**) is an inactive analog that retains the vinyl sulfone covalent warhead.

We have previously described the identification of (*E*)-5-(2-ethoxyphenyl)-*N*-(3-(methylsulfonyl)allyl)-1*H*-pyrazole-3-carboxamide (RA-0002034, **1**) as a covalent inhibitor of nsP2 cysteine proteases with potent antialphaviral activity (Figure 1).^8^ Although **1** met many of the criteria for a covalent chemical probe, it lacked chemical stability due to cyclization by an intramolecular aza-Michael reaction to an inactive cyclic dihydropyrazolo[1,5-*a*]pyrazin-4(5*H*)-one **2**,^9^ which occurred slowly at physiological pH and rapidly under basic conditions. Furthermore, commercial samples of **1** were often contaminated with varying levels of **2** that could only be quantified by NMR or extended HPLC runs.^9^ In this report, we describe the characterization of the isoxazole analog **3** that maintains potency for inhibition of nsP2pro, demonstrates remarkable proteome-wide selectivity, and has the chemical stability required of a high-quality chemical probe. We also describe a high-quality negative control analog **4** that retains the covalent warhead but has drastically reduced nsP2pro inhibition.

## 2. Results and Discussion

### 2.1 Chemical Probe Characterization

#### 2.1.1 Chemical Properties.

A structure-activity study of the heterocyclic core led to the identification of (*E*)-3-(2-ethoxyphenyl)-*N*-(3-(methylsulfonyl)allyl)isoxazole-5-carboxamide (RA-0003022, **3**), in which the pyrazole of **1** was replaced by a bioisosteric isoxazole that was no longer able to undergo intramolecular cyclization (Figure 1).^10^ Isoxazole **3** was isolated as a free flowing white solid with m.p. 178 °C that was stable when stored at room temperature for 6 months. Isoxazole **3** was also stable in solution across a pH range from 3–12 with no evidence of degradation or cyclization (Figure S1). Additional characterization of **3** showed that it had aqueous kinetic solubility of 20 μM, which was 500-fold above its IC_50_ for inhibition of nsP2pro,^10^ and showed no evidence of aggregation by dynamic light scattering (Figure S2). For measurement of intrinsic reactivity of the vinyl sulfone, **3** demonstrated a t_1/2_ of 70 min in the presence of 5 mM GSH (Figure S3), well above the minimum half-life recommended for covalent inhibitors.^7^

#### 2.1.2 Biological Activity.

**Figure 2.**
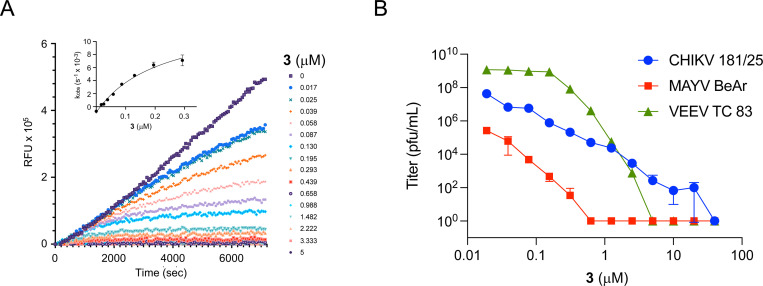
A: Isoxazole **3** inhibits CHIKV nsP2 protease activity in a time-dependent manner. Two-fold serial dilutions were used to determine *K*_i_, *k*_inact_, and *k*_inact_/*K*_i_. B: **3** demonstrated a 5–9 log decrease in viral titer against infectious isolates of CHIKV, MAYV, and VEEV alphaviruses. Data shown are averages ± SEM of two independent experiments.

RA-0003022 (**3**) was a potent covalent inhibitor of the CHIKV nsP2 protease with IC_50_ = 40 nM.^10^ To further characterize the kinetic properties of **3** as a covalent inhibitor, time dependent inactivation of CHIKV nsP2pro was measured at multiple enzyme concentrations (Figure 2A). Analysis of the kinetic inactivation data produced a *k*_inact_/*K*_i_ ratio of 6000 M^–1^s^–1^, demonstrating that isoxazole **3** was an efficient nonpeptide covalent cysteine protease inhibitor (Figure 2A). Isoxazole **3** also demonstrated potent inhibition of alphaviral replication using the CHIKV-nLuc and VEEV-nLuc reporter viruses with EC_50_ = 0.05 and 0.5 μM, respectively.^10^ To further profile **3** for potential pan-alphaviral activity it was tested for the reduction viral titer against infectious isolates of CHIKV, MAYV, and VEEV. Isoxazole **3** demonstrated a dose dependent 5–9 log decrease in viral titer (Figure 2B) against this range of New and Old World alphaviruses indicating its potential for pan-antialphaviral efficacy. Isoxazole **3** was also active when assayed against an EEEV-nLuc replicon (Figure S4). Importantly, **3** did not display cellular toxicity at concentrations up to 10 μM in human cells (A549ACE2 and HEK293) after 48h exposure (Figure S5).

#### 2.1.3 Target Engagement.

Mass spectrometry was performed to demonstrate target engagement of isoxazole **3** with the CHIKV nsP2pro active site catalytic cysteine. Purified nsP2pro was incubated with 1 μM of **3** in DMSO for 30 min, followed by tryptic digestion and LC-MS/MS analysis (n = 2). A single modification corresponding to the mass shift of vinyl sulfone **3** (+350.094) was identified at C478 on the A32-K38 (ANVCWAK) tryptic peptide (File S1 and Figure S6). This modification was only identified in the probe-treated samples and absent in the DMSO controls. No other nsP2pro cysteine residues were modified demonstrating the specificity for the active site catalytic C478 of CHIKV nsP2pro.

#### 2.1.4 Cysteine Protease Selectivity.

**Figure 3.**
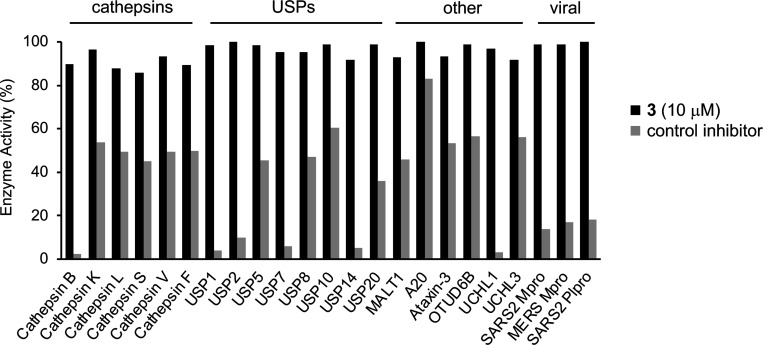
% Activity of isoxazole **3** at 10 μM against 20 human and 3 viral cysteine proteases. The following were used as controls: E-64 (50 nM) Cathepsin B, K, L, S, and V; Cystatin C (300 nM) for Cathepsin F; Z-VRPR-FMK (10 nM) for MALT1; Ubiquitin-Aldehyde (0.1 nM) for UCHL3, (1nM) for A20, UCHL1, USP1, USP14, (10 nM) for OTUD6B, USP2, USP5, USP7, USP8, USP10, USP20, and (1 μM) for Ataxin-3; GC376 (1 μM) for SARS2 Mpro; Protease Cocktail (10 μM) for MERS Mpro; and GRL0617 (10 μM) for SARS2 Plpro. Values are the average of duplicate determinations.

Isoxazole **3** was screened against a panel of 20 human cysteine proteases and three viral cysteine proteases at 10 μM in duplicate (Table S1). Compared to control inhibitors, **3** demonstrated no significant inhibition against this panel of cathepsins, ubiquitin specific peptidases (USPs), other human cysteine proteases, and viral papain-like cysteine proteases (Figure 3). The data indicated that β-aminomethyl vinyl sulfone **3** was >30-fold selective for CHIKV nsP2pro over the other cysteine proteases.

#### 2.1.5. Proteome-wide selectivity.

Isoxazole **3** was assessed in HEK293 cells by two independent chemoproteomics experiments: TAMRA labeling and biotin pulldown.

##### 2.1.5.1 Fluorescence-based chemoproteomics.

**Figure 4.**
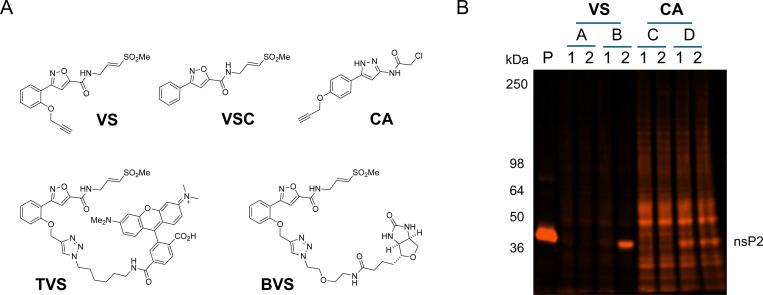
TAMRA fluorescence chemoproteomics. (A) Chemical structures of chemoproteomics probes **VS**, **VSC**, **CA**, **TVS**, and **BVS**. (B) Fluorescent imaging of SDS-PAGE following covalent labeling of purified nsP2 (lane P) by **TVS** or human cell lysates (lanes A–D) by **VS** or **CA**. In lanes 2, **VS** or **CA** were incubated with cell lysates prior to click reaction to append the TAMRA fluorophore. In lanes 1, prior to the click reaction, lysates were blocked with **VSC**. Lanes A and C contain HEK293 cell lysates (140 μg total protein each), whereas lanes B and D equivalent amounts of cell lysates were supplemented with purified CHIKV nsP2 (4 μg). The nsP2 band is marked. All ligands were used at 10 μM.

The selectivity of isoxazole **3** in human cells was evaluated by fluorescence-based chemoproteomics (Figure 4). Clickable isoxazole **VS** was synthesized, as well as the corresponding pre-clicked control (**TVS**) containing a TAMRA dye (Figure 4A). **VS** was a potent inhibitor of the CHIKV nsP2pro with IC_50_ = 65 nM and also demonstrated inhibition of CHIKV-nLuc replication with EC_50_ = 33 nM (Figure S7). As a positive control, the pre-clicked **TVS** demonstrated efficient labelling of purified full length nsP2 as indicated by fluorescence imaging of the expected band at 42.9 KDa (Figure 4B, lane P). Human HEK293 cell lysates were incubated with **VS** (10 μM, 30 min), followed by the sequence of click reaction with the TAMRA fluorophore, separation of proteins on a denaturing polyacrylamide gel, and fluorescence imaging. In the HEK293 cells lysate, no human proteins were observed to be labeled by **VS** following TAMRA click reaction (Figure 4B, lane A2). Pre-incubation of cell lysates with **TVS**, with addition of nsP2 protein, led to efficient TAMRA labeling of nsP2 (Figure 4B, lane B2) with no labelling of any other proteins from the HEK293 cell lysate. In a control reaction, no TAMRA labelling of nsP2 was observed when the active site cysteine was blocked by preincubation with **VSC** lacking an alkyne (Figure 4B, lane B2). As an additional positive control, clickable chloroacetamide **CA** was evaluated in the same TAMRA labelling experiments. Extensive labeling of the cell lysates was seen with **CA** (Figure 4B, lane C2), and pre-incubation with isoxazole **3** had no effect in reducing this labeling (Figure 4B, lane C1). Purified nsP2 was also labeled in spiked samples of the HEK293 cell lysate (Figure 4B, lane D2). Notably, pre-incubation with **VSC** did not result in reduced TAMRA labelling of the spiked nsP2 by **CA** (Figure 4B, lane D1), suggesting that labelling occurred on one or more of the 5 non-active site cysteines. These TAMRA labeling experiments demonstrated the selectivity of the vinyl sulfone covalent warhead in isoxazole **3** towards the nsP2pro catalytic cysteine, with the absence of labelling of other proteins in HEK293 cell lysate when compared to the relative promiscuity of chloroacetamide **CA**.

##### 2.1.5.2 HEK Lysate Pull-Down.

**Figure 5.**
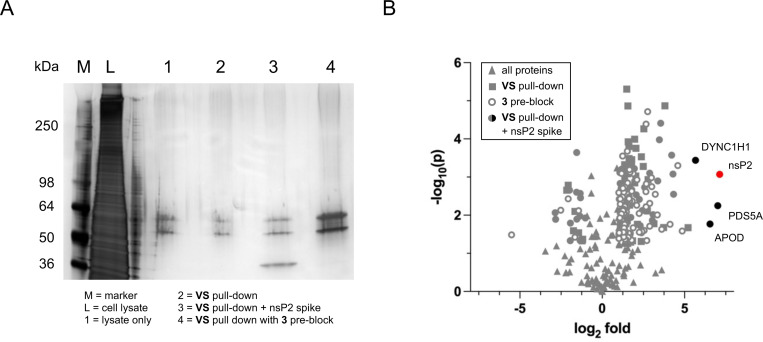
Streptavidin pull-down in HEK293 lysates by **VS** and biotin-azide click reaction. A: Silver stain of SDS-PAGE gel loaded with HEK293 lysate (lane L) or proteins from streptavidin pull-down of cell lysate alone (lane 1). The **VS** pull-down was performed 3 times from the lysate and loaded onto the gel: **VS** alone (lane 2), **VS** alone from lysate spiked with nsP2 protein (lane 3), VS from lysate pre-blocked with **3** (lane 4). B: Analysis of proteins identified from lanes 2–4 by LC-MS/MS. All identified proteins (▲); **VS** alone (■); **VS** after pre-block with **3** (❍); **VS** alone from lysate spiked with nsP2 protein (●). Proteins with log_2_ fold <5 enrichment are shown in grey. Proteins with log_2_ fold >5 enrichment are shown in black, with nsP2 in red.

To further investigate the human proteome-wide selectivity of isoxazole **3**, biotin-streptavidin pulldown experiments were conducted using HEK293 lysates to enrich for potential off-target proteins. Alkyne isoxazole **VS** was incubated with HEK293 cell lysates for 30 min followed by click reaction with biotin azide. The mixture was incubated with magnetic streptavidin beads for 15 min to capture biotin-labelled proteins. The beads were washed with 1% SDS, 8 M urea, and 20% acetonitrile to remove non-specific binding proteins. The enriched proteins were released from the streptavidin beads by heating in SDS buffer. The proteins were run on a denaturing polyacrylamide gel and silver staining did not indicate any highly enriched proteins (Figure 5A, lane 2 vs. 1), further supporting the apparent high selectivity of isoxazole **3** against the proteins in the HEK293 cell lysate. To further analyze for potential off-target proteins, lanes 1 and 2 were subjected to trypsin digestion and LC-MS/MS analysis. Proteins were identified and quantified using MaxQuant software and the resulting list was filtered to remove common contaminates from sample handling. Using an enrichment cut-off of log_2_ fold-change >2 (lane 2 over 1) and number of unique peptides >2,^11^ a total of 14 proteins were identified representing candidate off-targets and non-specific proteins that were captured by the streptavidin beads (File S2). To account for non-specific proteins captured by the beads, a parallel biotin-streptavidin pull down experiment was performed using cell lysate that was pre-incubated with isoxazole **3** (10 μM) for 30 min prior to addition of **VS** (Figure 5A, lane 4). This control experiment indicated that proteins with up to 5-fold enrichment in comparison to the cell lysate alone (lane 4 over 1) could be designated non-specific binding proteins, including all 14 of the candidate off-targets (Figure 5B). A third biotin-streptavidin pull down was performed using HEK293 cell lysate that was spiked with purified nsP2 protein (4 μg) as a positive control (Figure 5A, lane 3). In this experiment, LC-MS/MS analysis identified the control nsP2 protein was enriched 7-fold and three candidate off-target proteins with >5-fold enrichment: a dynein heavy chain (DYNC1H1), a chromatid cohesion protein (PDS5A), and an apolipoprotein (APOD). Notably, none of these three candidate off-targets were identified in the biotin-streptavidin pull down with **VS** alone (Figure 5A, lane 2), indicating that they are also unlikely to be specific off-targets of the vinyl sulfone. The combined results (Figure 5B) demonstrate the remarkable proteome-wide selectivity of the vinyl sulfone in isoxazole **3**, with no human cysteine proteases or other enzymes identified as off-targets.

### 2.2. Negative Control Analog

**Figure 6.**
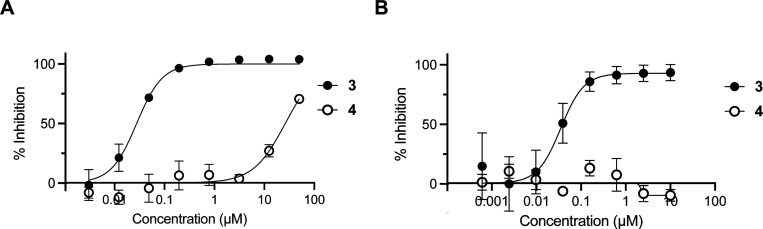
Dose-response curves of isoxazole **3** and negative control analog **4**. A: Inhibition of CHIKV nsP2pro following a 30 min incubation. Values are the mean of triplicate determinations. B: Inhibition of CHIKV-nLuc replication in human fibroblast MRC5 cells at 6 h post-inoculation with the virus. Values are the average of duplicate determinations.

The methoxymethyl (MOM) derivative of pyrazole **1** (MOM-**1**, Figure S8) had been nominated previously as a negative control of pyrazole **1**.^8^ However, MOM-**1** demonstrated residual inhibition of CHIKV nsP2pro at 10 μM and robust inhibition of CHIKV-nLuc replication at 1 μM that severely limited its utility as a negative control in cells. MOM-**1** contained an acid labile protecting group that may degrade to generate the potent nsP2 inhibitor pyrazole **1**, which may be one reason for its antiviral activity in cells. Thus, we sought to identify a high-quality negative control analog of **3** with improved chemical stability and without residual nsP2pro inhibition. Analysis of the structure-activity relationships for inhibition of CHIKV nsP2pro^10^ identified (*E*)-3-(2-ethoxyphenyl)-*N*-isopropyl-*N*-(3-(methylsulfonyl)allyl)isoxazole-5-carboxamide (RA-0025453, **4**) as a candidate negative control compound (Figure 1). Isoxazole **4** contained an isopropyl substituent on the amide nitrogen but retained the critical vinyl sulfone covalent warhead. In enzyme assays, **4** demonstrated very weak CHIKV nsP2pro inhibition with >100-fold reduction in potency compared to isoxazole **3** (Figure 6A). In addition, the isopropyl-substituted analog **4** was fully inactive in the CHIKV-nLuc replication assay at concentrations up to 10 μM. Isoxazole **4** was stable upon prolonged storage and in solution across a wide range of pH (Figure S1), and thus meets the criteria of a high-quality negative control analog for the chemical probe **3**.

### 2.3 Chemistry

#### 2.3.1 Chemical Probe and Negative Control.

**Scheme 1.**
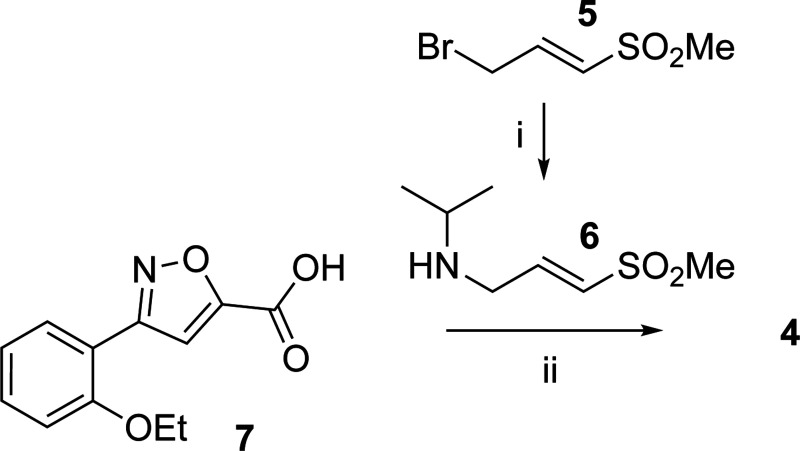
Synthesis of Negative Control **4**^*a*^ ^*a*^Reagents and conditions: (i) propan-2-amine, Cs_2_CO_3_; (ii) TBTU, DIPEA.

Synthesis of isoxazole **3** was accomplished as previously reported.^10^ Negative control analog **4** was synthesized in two steps starting from (*E*)-3-bromo-1-(methylsulfonyl)prop-1-ene **5**. Substitution of the bromine by 2-propanamine afforded (*E*)-*N*-isopropyl-3-(methylsulfonyl)prop-2-en-1-amine **6**, which was followed by amide coupling with **7** to yield (*E*)-3-(2-ethoxyphenyl)-*N*-isopropyl-*N*-(3-(methylsulfonyl)allyl)isoxazole-5-carboxamide **4** (Scheme 1).

#### 2.3.2 Analogs for Chemoproteomics.

**Scheme 2.**
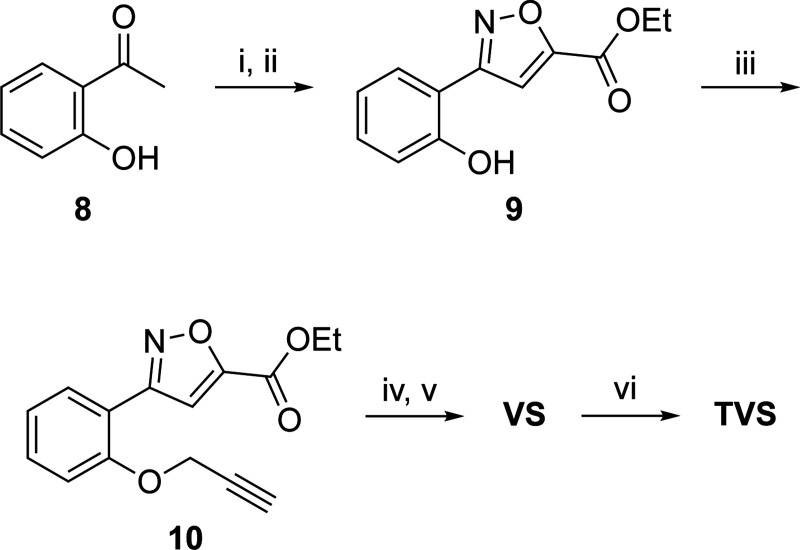
Synthesis of clickable vinyl sulfone (**VS**) and pre-clicked vinyl sulfone control (**TVS**)^*a*^ ^*a*^Reagents and conditions: (i) diethyl oxalate, NaH; (ii) NH_2_OH.HCl; (iii) 3-bromoprop-1-yne, K_2_CO_3_; (iv) NaOH; (v) (*E*)-3-(methylsulfonyl)prop-2-en-1-amine, TBTU, pyridine; (vi) 6-TAMRA azide, CuSO_4_, Na ascorbate.

The clickable derivative **VS** containing an alkyne handle was synthesized by modification of the previously described methods (Scheme 2).^10^ Claisen condensation of acetophenone **8** with diethyl oxalate in presence of sodium hydride followed by cyclization with hydrazine hydrate under acidic condition yielded the isoxazole **9**. Incorporation of the alkyne handle was achieved by propargylation of the hydroxy group to give **10**. Saponification and amide coupling yielded the clickable derivative **VS**. A control analog **VSC** lacking the clickable alkyne group was synthesized by amide coupling reaction of the commercially available unsubstituted phenyl analog with the vinyl sulfone warhead. The pre-clicked control **TVS** was synthesized by reaction of **VS** with the TAMRA dye using a Cu-catalyzed azide-alkyne [3 + 2] cycloaddition reaction (Scheme 2). Clickable chloroacetamide **(CA)** was synthesized by the previously described method.^8^

### 2.4 Discussion

In this study, we describe the characterization of isoxazole **3** as a high-quality covalent chemical probe for CHIKV nsP2pro. Isoxazole **3** meets all of the state-of-the-art requirements of a covalent chemical probe including fast kinetics of enzyme inactivation, proteome-wide selectivity, and potent cellular activity without toxicity.^7^ Notably, vinyl sulfone **3** demonstrated pan-antialphaviral activity and will be a powerful tool to study the role of nsP2pro in the replication, propagation, and virulence of New and Old World alphaviruses that remain a global public health threat.^3, 12^ A negative control analog **4** was identified that retained the structural features of the chemical probe, including the vinyl sulfone covalent warhead, but demonstrated >100-fold reduction in potency against CHIKV nsP2pro and was inactive in antiviral assays. The β-aminomethyl vinyl sulfone **3** demonstrated remarkable selectivity for the nsP2 cysteine protease and showed no activity against a large panel of cathepsins, ubiquitin specific peptidases, other human cysteine proteases, and viral Papain-like proteases. Chemoproteomic profiling from HEK293 cells using orthogonal techniques further demonstrated the remarkable proteome-wide selectivity profile of **3**.

Vinyl sulfones are often listed as highly reactive covalent warheads that are promiscuous cysteine electrophiles ^13, 14^. However, there is a distinct difference in electrophilicity between internal vinyl sulfones as found in **1** and **3** and external vinyl sulfones which are found in many of the promiscuous electrophiles. Three internal vinyl sulfones are currently under clinical development: K777, a parasite cysteine protease inhibitor^15^; VVD-133214, a WRN helicase inhibitor^16^; and rigosertib, a multi-kinase inhibitor targeting PI3K and PLK.^17^ In contrast, no external vinyl sulfone is listed in clinical development. K777, a peptidyl-styryl sulfone, demonstrated selective targeting of cathepsin B and L in Vero E6 cells.^18^ VVD-133214, a methyl vinyl sulfone, selectively engaged C727 located in the helicase domain of WRN and demonstrated proteome-wide selectivity over other cellular proteins.^16^ Rigosertib, a benzyl styryl sulfone, was a selective and non-ATP-competitive PLK1 and PI3K inhibitor^19^ but also interacted with RAS signaling proteins^20^ and its full proteome selectivity data has not been reported. We now add the nsP2 cysteine protease inhibitors **1** and **3** as examples of internal vinyl sulfones that act as covalent modifiers of their target enzyme with high proteome-wide selectivity. These examples demonstrate that internal vinyl sulfones are warheads that warrant additional study as chemotypes for development of covalent chemical probes and candidate drug molecules. ^13^

In summary, RA-0003022 (**3**) is a high-quality chemical probe and RA-0025453 (**4**) is a negative control analog that can be used to study the role of the nsP2pro in alphaviral replication and virulence. It meets the full range of biological and chemical criteria^7^ for a high-quality chemical probe (Table 1).

## 3. Methods

### 3.1 General Chemistry Methods.

All reactions were conducted in oven-dried glassware under a dry nitrogen atmosphere unless otherwise specified. All reagents and solvents were obtained from commercial sources and used without further purification. No unexpected safety hazards were encountered during the synthesis. Analytical thin layer chromatography (TLC) was performed on pre-coated silica gel plates (200 μm, F_254_ indicator), visualized under UV light or by staining with iodine and KMnO_4_. Column chromatography utilized pre-loaded silica gel cartridges on a Biotage automated purification system. ^1^H and ^13^C NMR spectra were recorded in DMSO-d_6_ and CD_3_CN at 400/500/700 and 101/126/176 MHz, respectively, on a Bruker spectrometer. Chemical shifts (*δ*) are reported in parts per million (ppm) downfield from tetramethylsilane for ^1^H NMR, with major peaks designated as s (singlet), d (doublet), t (triplet), q (quartet), and m (multiplet). High-resolution mass spectrometry (HRMS) analyses were performed at the UNC Department of Chemistry Mass Spectrometry Core Laboratory using a Q Exactive HF-X mass spectrometer. Liquid chromatography-mass spectrometry (LC-MS) was conducted on an Agilent 1290 Infinity II LC System with an Agilent Infinity Lab PoroShell 120 EC-C18 column (30 °C, 2.7 μm, 2.1 × 50 mm), employing a 5−95% CH₃CN in water eluent, with 0.2% (v/v) formic acid as the modifier and a flow rate of 1 mL/min. Preparative high-performance liquid chromatography (HPLC) was executed using an Agilent 1260 Infinity II LC System equipped with a Phenomenex C18 column (PhenylHexyl, 30 °C, 5 μm, 75 × 30 mm), with a 5−95% CH₃CN in water eluent and 0.05% (v/v) trifluoroacetic acid as the modifier, at a flow rate of 30 mL/min. Analytical HPLC data were recorded on a Waters Alliance HPLC with a PDA detector or an Agilent 1260 Infinity II series with a PDA detector (EC-C18, 100 mm × 4.6 mm, 3.5 μm), using a 10−90% CH₃CN in water eluent at a flow rate of 1 mL/min. The final compounds were confirmed to be >95% pure by HPLC analysis. Isoxazole **3**, 3-(2-ethoxyphenyl)isoxazole-5-carboxylic acid (**7**), and clickable chloroacetamide **CA** were synthesized following the reported procedures.^8, 10^

### 3.2 (E)-3-(2-ethoxyphenyl)-N-isopropyl-N-(3-(methylsulfonyl)allyl)isoxazole-5-carboxamide (4).

To a stirred solution of (*E*)-3-bromo-1-(methylsulfonyl)prop-1-ene (**5**, 0.5 g, 2.5 mmol, 1.0 eq.) in DCM (10 mL) were added propan-2-amine (0.1 g, 0. mmol, 1.0 eq.) and Cs_2_CO_3_ (0.3 g, 0.9 mmol, 1.5 eq.) at 0 °C and stirred at 25 °C for 2 h. On completion of the reaction based on TLC and LCMS, the mixture was filtered through celite bed and concentrated under vacuum to afford crude (*E*)-*N*-isopropyl-3-(methylsulfonyl)prop-2-en-1-amine (**6**) (0.4 g, 92% yield) as a colorless liquid. MS (ESI) *m/z*: 178.2 [M+H]^+^.

To a stirred solution of carboxylic acid **7** (0.25 g, 1.07 mmol, 1.0 eq.) in DMF (5.0 mL) were added amine **6** (0.2 g, 1.3 mmol, 1.2 eq.), TBTU (0.4 g, 1.3 mmol, 1.2 eq.), DIPEA (0.4 mL, 2.1 mmol, 2.0 eq.) and the reaction was stirred at 25 °C for 2 h. On completion of the reaction based on TLC and LCMS, the mixture was diluted with water (30 mL), organic layer extracted with EtOAc (3 × 50 mL), washed with brine, dried over anhydrous Na_2_SO_4_, filtered, and concentrated. The resulting crude was purified by preparative HPLC to afford (*E*)-3-(2-ethoxyphenyl)-*N*-isopropyl-*N*-(3-(methylsulfonyl)allyl)isoxazole-5-carboxamide (**4**) (0.05 g, 12%) as a white sticky solid. ^1^H NMR (400 MHz, DMSO-*d*_6_): *δ* 7.89 (d, *J* = 7.7 Hz, 1H), 7.55 – 7.45 (m, 1H), 7.22 (d, *J* = 8.4 Hz, 1H), 7.12 (t, *J* = 7.6 Hz, 1H), 7.02 (s, 1H), 6.80 (s, 2H), 4.25 (q, *J* = 7.0 Hz, 5H), 2.99 (s, 3H), 1.44 (t, *J* = 7.0 Hz, 3H), 1.22 (d, *J* = 6.6 Hz, 6H). ^13^C NMR (101 MHz, DMSO-*d*_6_): *δ* 165.9, 160.8, 159.3, 155.4, 142.4, 132.4, 131.1, 127.2, 120.8, 114.7, 112.9, 103.2, 64.1, 50.1, 42.2, 40.6, 20.8, 19.7, 14.5. HRMS (ESI) *m/z*: [M+H]^+^ calculated for C_19_H_25_N_2_O_5_S: 393.1484, found 393.1481. HPLC purity (254 nm) >99%.

### 3.3 (E)-N-(3-(methylsulfonyl)allyl)-3-(2-(prop-2-yn-1-yloxy)phenyl)isoxazole-5-carboxamide (VS).

To a stirred solution of 1-(2-hydroxyphenyl)ethan-1-one (**8**) (5 g, 36.8 mmol, 1.0 eq.) in toluene (200 mL) was added NaH (7.4 g, 184.1 mmol, 5.0 eq.) portion-wise at 0 °C, and the reaction was stirred at 25 °C for 0.5 h. Then diethyl oxalate (7.5 mL, 55.2 mmol, 1.5 eq.) was added and the reaction was stirred at 60 °C for 2 h. On completion of the reaction based on TLC and LCMS analysis, the reaction was poured into water (200 mL), quenched by 1 N HCl solution till pH ~ 3, extracted with EtOAc (2 × 200 mL), combined organic layers dried over anhydrous Na_2_SO_4_, filtered, and concentrated. The resulting crude was purified by combiflash (eluted with 20% EtOAc in hexane) to afford ethyl 4-(2-hydroxyphenyl)-2,4-dioxobutanoate (4.6 g, 57% yield) as colorless oil. MS (ESI) *m/z*: 237.4 [M+H]^+^. The product was dissolved in EtOH (50 mL), hydroxylamine hydrochloride (2.7 g, 38.9 mmol, 2.0 eq.) was added and the reaction was stirred at 90 °C for 6 h. On completion of the reaction based on TLC and LCMS, the mixture was diluted with water (200 mL), organic layer extracted with EtOAc (2 × 200 mL), washed with brine, dried over anhydrous Na_2_SO_4_, filtered, and concentrated. The resulting crude was purified by combiflash (eluted with 10 % EtOAc in hexane) to afford ethyl 3-(2-hydroxyphenyl)isoxazole-5-carboxylate (**9**) (1.4 g, 30% yield) as a white solid. MS (ESI) *m/z*: 234.4 [M–H]^–^.

To a stirred solution of **9** (1.4 g, 6.0 mmol, 1.0 eq.) in DMF (14 mL) were added K_2_CO_3_ (1.7 g, 12.0 mmol, 2.0 eq.) and 3-bromoprop-1-yne (0.9 g, 7.2 mmol, 1.2 eq.). The reaction mixture was stirred at 25 °C for 16 h. On completion of the reaction based on TLC and LCMS, the mixture was diluted with water (10 mL), organic layer extracted with EtOAc (2 × 10 mL), washed with brine, dried over anhydrous Na_2_SO_4_, filtered, and concentrated. The resulting crude was purified by combiflash (eluted with 30 % EtOAc in hexane) to afford ethyl 3-(2-(prop-2-yn-1-yloxy)phenyl)isoxazole-5-carboxylate (**10**) (0.7 g, 43% yield) as white solid. MS (ESI) *m/z*: 272.1 [M+H]^+^.

To a stirred solution of **10** (0.7 g, 2.6 mmol, 1.0 eq.) in MeOH (7 mL) were added NaOH (0.3 g, 5.2 mmol, 2.0 eq.) and water (4 mL). The reaction mixture was stirred at 25 °C for 1 h. On completion of the reaction based on TLC and LCMS, the reaction mixture was diluted with water (20 mL), acidified by 1N HCl till pH ~3, extracted with DCM (2 × 20 mL), combined organic layers dried over anhydrous Na_2_SO_4_, filtered, and concentrated to afford 3-(2-(prop-2-yn-1-yloxy)phenyl)isoxazole-5-carboxylic acid (0.6 g, 88% yield). MS (ESI) *m/z*: 244.2 [M–H]^−^. To a stirred solution of this carboxylic acid (0.2 g, 0.8 mmol, 1.0 eq.) in pyridine (2.0 mL) were added (*E*)-3-(methylsulfonyl)prop-2-en-1-amine^10^ (0.1 g, 0.8 mmol, 1.0 eq.), TBTU (0.4 g, 1.2 mmol, 1.5 eq.) and the reaction was stirred at 25 °C for 1 h. On completion of the reaction based on TLC and LCMS, the mixture was diluted with water (10 mL), organic layer extracted with EtOAc (2 × 10 mL), washed with brine, dried over anhydrous Na_2_SO_4_, filtered, and concentrated. The resulting crude was purified by combiflash (eluted with 50% EtOAc in hexane), followed by preparative HPLC to afford *(E)-N-(3-(methylsulfonyl)allyl)-3-(2-(prop-2-yn-1-yloxy)phenyl)isoxazole-5-carboxamide* (**VS**) (0.08 g, 27% yield) as a white solid: m.p. 173 °C. ^1^H NMR (400 MHz, DMSO-*d*_6_): *δ* 9.24 (t, *J* = 5.8 Hz, 1H), 7.90 (dd, *J* = 8.1, 1.7 Hz, 1H), 7.62 (ddd, *J* = 8.7, 7.2, 1.7 Hz, 1H), 7.44 (dd, *J* = 8.5, 1.2 Hz, 1H), 7.36 (ddd, *J* = 8.3, 7.2, 1.2 Hz, 1H), 7.18 (s, 1H), 6.80 (d, *J* = 1.6 Hz, 2H), 4.82 (d, *J* = 2.4 Hz, 2H), 4.14 (dd, *J* = 5.9, 2.4 Hz, 2H), 3.51 (t, *J* = 2.4 Hz, 1H), 3.00 (s, 3H). ^13^C NMR (100 MHz, DMSO-*d*_6_): 159.5, 150.7, 148.2, 143.1, 142.1, 131.8, 130.5, 125.7, 122.6, 118.1, 117.0, 98.2, 80.3, 77.5, 61.6, 42.1, 39.2. HRMS (ESI) *m/z*: [M+H]^+^ calculated for C_17_H_17_N_2_O_5_S: 361.0858, found 361.0845. HPLC purity (254 nm) >99%.

### 3.4 (E)-N-(9-(2-carboxy-4-((6-(4-((2-(5-((3-(methylsulfonyl)allyl)carbamoyl)isoxazol-3-yl)phenoxy)methyl)-1H-1,2,3-triazol-1-yl)hexyl)carbamoyl)phenyl)-6-(dimethylamino)-3H-xanthen-3-ylidene)-N-methyl methanaminium (TVS).

**VS** (5.5 mg, 0.015 mmol, 1.0 eq.) was mixed with 6-TAMRA azide (10.0 mg, 0.018 mmol, 1.0 eq.), CuSO_4_.5H_2_O (0.4 mg, 0.0015 mmol, 0.1 eq.), and Na-ascorbate (0.6 mg, 0.003 mmol, 0.2 eq.) in DMSO (2.0 mL) for 1 h at 25 °C. On completion of the click reaction based on TLC and LCMS, solvent was removed in vacuo and the crude was purified by preparative HPLC to afford the desired pre-clicked vinyl sulfone (**TVS**) as a pink oil (5.0 mg, 36% yield). ^1^H NMR (700 MHz, CD_3_CN): *δ* 8.30 (d, *J* = 8.3 Hz, 1H), 8.08 (dd, *J* = 8.3, 1.8 Hz, 1H), 7.94 – 7.89 (m, 2H), 7.82 – 7.74 (m, 2H), 7.52 (ddd, *J* = 8.7, 7.2, 1.6 Hz, 1H), 7.38 (t, *J* = 5.9 Hz, 1H), 7.35 – 7.26 (m, 2H), 7.13 – 7.05 (m, 3H), 6.87 (dd, *J* = 9.5, 2.5 Hz, 2H), 6.79 (dt, *J* = 15.2, 4.5 Hz, 1H), 6.75 (d, *J* = 2.4 Hz, 2H), 6.57 (dt, *J* = 15.2, 1.9 Hz, 1H), 5.17 (s, 2H), 4.30 (t, *J* = 6.8 Hz, 2H), 4.09 (ddd, *J* = 6.2, 4.5, 1.9 Hz, 2H), 3.29 (q, *J* = 6.5 Hz, 2H), 3.21 (s, 10H), 2.86 (s, 3H), 2.51 (s, 1H), 1.83 (p, *J* = 7.1 Hz, 2H), 1.48 (p, *J* = 7.0 Hz, 2H), 1.34 – 1.28 (m, 3H), 1.27 – 1.21 (m, 3H). ^13^C NMR (176 MHz, CD_3_CN): *δ* 166.6, 166.2, 160.9, 160.0, 158.4, 158.3, 151.9, 148.7, 144.7, 143.9, 143.6, 139.5, 135.1, 133.9, 132.5, 131.9, 131.4, 129.7, 129.6, 126.6, 124.9, 123.8, 119.8, 118.8, 116.6, 115.2, 114.4, 99.7, 97.2, 68.5, 50.6, 42.9, 41.3, 41.2, 40.3, 40.1, 30.6, 29.8, 26.6, 26.5. HRMS (ESI) *m/z*: [M+H]^+^ calculated for C_48_H_52_N_8_O_9_S: 916.3572, found 916.3529. HPLC purity (254 nm) >99%.

### 3.5 (E)-N-(3-(methylsulfonyl)allyl)-3-phenylisoxazole-5-carboxamide (VSC).

To a stirred solution of 3-phenylisoxazole-5-carboxylic acid (0.2 g, 1.1 mmol, 1.0 eq.) and TBTU (0.5 g, 1.6 mmol, 1.5 eq.) in pyridine (5 mL) was added (*E*)-3-(methylsulfonyl)prop-2-en-1-amine^10^ (0.18 g, 1.1 mmol, 1.0 eq.) and the reaction was stirred at 25 °C for 2 h. On completion of the reaction (based on TLC and LCMS analysis), the reaction was poured into water, extracted with EtOAc, combined organic layers dried over anhydrous Na_2_SO_4_, filtered, and concentrated. The crude was purified by preparative HPLC to afford (*E*)-*N*-(3-(methylsulfonyl)allyl)-3-phenylisoxazole-5-carboxamide (**VSC**) as a white solid (0.15 g, 34% yield,): m.p 164 °C. ^1^H NMR (500 MHz, DMSO-*d*_6_): *δ* 9.39 (t, *J* = 5.8 Hz, 1H), 7.96 – 7.91 (m, 2H), 7.69 (s, 1H), 7.55 (dt, *J* = 4.6, 2.8 Hz, 3H), 6.86 – 6.77 (m, 2H), 4.15 (dd, *J* = 5.7, 3.1 Hz, 2H), 3.01 (s, 3H). ^13^C NMR (126 MHz, DMSO-*d*_6_): *δ* 163.9, 162.6, 155.7, 142.1, 130.7, 130.5, 129.3, 127.8, 126.7, 104.9, 42.1, 38.9. HRMS (ESI) *m/z*: [M+H]^+^ calculated for C_14_H_15_N_2_O_4_S: 307.0753, found 307.0741. HPLC purity (254 nm) >99%.

### 3.6 General Biological Methods.

The CHIKV nsP2 protease, CHIKV-nLuc viral replication, VEEV-nLuc viral replication, and alphavirus (CHIKV, MAYV, and VEEV) titer reduction assays were performed using the reported protocols.^8^

### 3.7 EEEV-nLuc Replicon Assay.

To generate an EEEV-nLuc replicon plasmid construct (pEEEV-V105-nLuc-Rep), the EEEV strain V105 cDNA sequence (GenBank KP282670), encoding the nanoluciferase (nLuc) gene sequence (GenBank AFI79295) in place of the viral structural genes, was cloned downstream of the 26S subgenomic promoter and upstream of the 3’ UTR in a manner identical to the viral structural gene open reading frame. The EEEV V105 cDNA sequence was modified to match type-specific consensus sequences in the 5’ and 3’ UTRs that were missing from the strain sequence as previously described.^21^ EEEV-V105-nLuc replicon RNA encoding the viral nonstructural proteins and the nLuc gene was synthesized by in vitro transcription by mMessage mMachine SP6 transcription (Invitrogen; AM1340) using linearized, sequence-confirmed pEEEV-V105-nLuc-Rep plasmid as a template. Proper synthesis of viral replicon RNA was confirmed by gel electrophoresis and the RNA concentration was measured by UV spectrometry. Low-passage normal human dermal fibroblasts (ATCC PCS-201–012) that had been maintained at 37 °C and 5% CO_2_ in Dulbecco’s Modified Eagle medium (Gibco; 11965–084) supplemented with 10% fetal bovine serum and 100 U/mL penicillin-streptomycin were seeded into black 96-well plates (Greiner Bio-One; 655086) at a density of 5.7 × 10^3^ cells per well in a volume of 100 μl and cultured overnight. The next day, 1 μg of EEEV-V105-nLuc replicon RNA was transfected into the cells using Lipofectamine MessengerMAX Reagent (Invitrogen; LMRNA015) according to the manufacturer’s instructions. Following RNA transfection, the transfection medium was removed and replaced with culture medium supplemented with 2-fold serial dilutions of **3** (0.16–20 μM). Control cells were maintained in an equal volume of culture medium supplemented with DMSO. At 24 h post-treatment, nLuc activity was quantified using the Nano-Glo Luciferase Assay System (Promega; N1120) according to the manufacturer’s instructions. Luminescence was measured on a Tecan infinite M plex plate reader with automatic attenuation luminescence setting and 1000 ms integration time.

### 3.8 Cysteine Protease Selectivity Assays.

Cysteine protease inhibition assays were performed in duplicate at BPS Bioscience Inc., San Diego, CA. A solution of **3** ten-fold higher than the final concentration was prepared with 10% DMSO in assay buffer and 5 μL of the dilution was added to a 50 μL reaction so that the final concentration of DMSO was 1% in all of the reactions. All control samples, including background and no compound controls, also contain 1% DMSO. The compounds were pre-incubated in duplicate at room temperature for 30 min in a mixture containing assay buffer, enzyme, and isoxazole **3**. For cathepsin assays, the enzymatic reactions were conducted in duplicate at room temperature for 30 min in a 100 μL mixture containing 50 mM MES buffer, pH 5.0, 100 mM NaCl, 5 mM DTT, a cathepsin substrate, a cathepsin enzyme, and isoxazole **3**. Fluorescence intensity was measured at an excitation of 360 nm and an emission of 460 nm using a Tecan Infinite M1000 microplate reader. For cathepsin D and E, fluorescence intensities were measured at an excitation of 330 nm and an emission of 390 nm using a Tecan Infinite M1000 microplate reader. For deubiquitinase assays, the enzymatic reactions were conducted in duplicate at room temperature for 30 min in a 50 μL mixture containing 50 mM Tris-HCl, pH 7.4, 0.5 mM EDTA, 0.05% Tween 20, 1 mM DTT, 100 nM Ubiquitin-AMC substrate, a deubiquitinase enzyme, and isoxazole **3**. Fluorescence intensity was measured at an excitation of 360 nm and an emission of 460 nm using a Tecan Infinite M1000 microplate reader. For MALT1 assay, the MALT1 and probe compound **3** were pre-incubated for 30 min at room temperature. Then, the enzymatic reactions were begun in duplicate with the addition of the substrate at room temperature for 30 min. This reaction contained a 50 μL mixture containing 50 mM HEPES, pH 8.0, 150 mM NaCl, 1 M Na Citrate, 0.05% CHAPS, 20 μM Ac-LRSR-AMC, MALT1 enzyme, and probe compound **3**. For the Papain-like protease (SARS-CoV-2) the assay kit was pre-incubated in duplicate at 37°C, while for the 3CL Protease (MERS-CoV) and 3CL Protease (B.1.1.529, Omicron Variant) (SARS-CoV-2) the assay kits were pre-incubated in duplicate at room temperature for 30 min in a mixture containing assay buffer, enzyme, and **3**. After 30 min, the enzymatic reactions were initiated by the addition of the substrate. The enzymatic reaction proceeded for 30 min to 4 h at either 37 °C or room temperature, respectively. Fluorescence intensity was measured at an excitation of 360 nm and an emission of 460 nm using a Tecan Infinite M1000 microplate reader. The fluorescent intensity data were analyzed using the computer software, Graphpad Prism. In the absence of **3**, the fluorescent intensity (Ft) in each data set was defined as 100% activity. In the absence of the enzyme, the fluorescent intensity (Fb) in each data set was defined as 0% activity. The percent activity in the presence of each compound was calculated according to the following equation: % activity = (F-Fb)/(Ft-Fb), where F = the fluorescent intensity in the presence of **3**.

### 3.9 nsP2 Target Engagement.

5 μL of purified nsP2 protease (2 mg/mL) was diluted in 93 μL of reaction buffer (25 mM HEPES pH 7.3, 1 mM DTT, 1% DMSO). 2 μL of a 50 μM stock solution in buffer of isoxazole **3** (final concentration 1 μM) or 2 μL of DMSO (prepared in the same dilutions as for the compound) was added and the mixture incubated at 24 °C for 30 min. 100 μL of each sample were reduced with 5 mM DTT at 37°C for 45 mins and alkylated with 15 mM iodoacetamide in the dark for 45 mins. Samples were digested with trypsin (Promega, 1:20 w/w) overnight at 37 °C. The resulting peptide samples were acidified to 0.1% trifluoracetic acid, desalted using C18 spin columns (Pierce), and the eluates were dried via lyophilization. The samples were analyzed by LC-MS/MS using an Easy nLC 1200 coupled to a QExactive HF mass spectrometer (Thermo Scientific). Samples were injected onto an Easy Spray PepMap C18 column (75 μm id × 25 cm, 2 μm particle size) (Thermo Scientific) and separated over a 120 min method. The gradient for separation consisted of 5–38% mobile phase B at a 250 nL/min flow rate, where mobile phase A was 0.1% formic acid in water and mobile phase B consisted of 0.1% formic acid in 80% ACN. The QExactive HF was operated in data-dependent mode where the 15 most intense precursors were selected for subsequent fragmentation. Resolution for the precursor scan (*m/z* 350–1600) was set to 60,000 with a target value of 3 × 10^6^ ions. MS/MS scans resolution was set to 15,000 with a target value of 1 × 10^5^ ions, 100 ms injection time. The normalized collision energy was set to 27% for HCD. Dynamic exclusion was set to 30 s, peptide match was set to preferred, and precursors with unknown charge or a charge state of 1 and ≥ 8 were excluded. Raw data was first processed using PMi-Byonic (Protein Metrics, version 5.1.1) with an open search to detect any unexpected deviation from the hypothesized mass shift corresponding to the isoxazole **3** (+351.10147 Da). Files were searched against the CHIKV-nsP2 sequence, and a wildcard open search was selected with a mass shift range of 250–450 Da mass shift on cysteine residues. Results were filtered for > 250 PSM Score, < 0.001 protein-aware posterior error probability, and > 10 modification score. A high-confidence mass shift of +350.094, likely corresponding to the isoxazole **3**, was detected in the treated samples but not the controls. Data was then processed using Proteome Discoverer (Thermo Scientific, version 3.1) and searched against a reviewed Uniprot *E.coli* BL21 database (containing 4,156 sequences; downloaded April 2023), appended with the CHIKV-nsP2 sequence, and a common contaminants database (MaxQuant, 245 sequences), using the Sequest HT search algorithm within Proteome Discoverer. Enzyme specificity was set to trypsin, up to two missed cleavage sites were allowed; Isoxazole **3** (+351.10147, +350.094 Da) and carbamidomethylation of Cys, as well as oxidation of Met were set as variable modifications. A precursor mass tolerance of 10 ppm and fragment mass tolerance of 0.02 Da were used. Label-free quantification (LFQ) was enabled. Peptides were filtered based on a 1% false discovery rate (FDR). PSM fragment ion data was exported from Proteome Discoverer and inputted into the Interactive Peptides Spectral Annotator tool.^22^

### 3.10 Fluorescence-based Chemoproteomics.

HEK293 cells were lysed by sonication and the resulting lysates (~3.5 mg/mL) diluted in reaction buffer (25 mM HEPES pH 7.3, 1 mM DTT, 1% DMSO). 2 μL of a 250 μM stock solution in DMSO of clickable vinyl sulfone (**VS**) or clickable chloroacetamide (**CA**) were added to 40 μL of cell lysate (~140 μg total protein) and the mixtures were incubated at 24 °C for 30 min. Equal volumes of THPTA (5 mM stock in H_2_O) and CuSO_4_.H_2_O (10 mM stock in H_2_O) were premixed to generate the ligand-copper solution. For the click reaction, freshly prepared solutions of 10 μL 6-TAMRA azide (50 μM stock in buffer), 20 μL ligand-copper premix, and 10 μL sodium ascorbate (10 mM stock in H_2_O) were added successively to cell lysates, and the mixture was incubated in the dark at 24 °C for 1 h. On completion, 45 μL of the reaction was mixed with 45 μL of SDS-PAGE sample buffer [1:5 volumes of 10x reducing agent (NuPAGE; cat. NP0009) to 2X SDS sample buffer (Novex, cat. LC2676)] and heated at 85 °C for 5 min. Samples were separated in a 4–12% Tris-Glycine SDS-PAGE (Novex, catalog number XP04125BOX). The TAMRA labeling was visualized on an Invitrogen iBrightFL1000 (515–545 nm excitation; 568–617 nm emission). Total protein was visualized following Coomassie staining. For reactions containing **VSC**, the competitor compound was added to the cell lysate (2 μL of a 240 μM stock solution in DMSO, final concentration 10 μM) and incubated at 24 °C for 30 min prior to the addition of the **VS** or **CA**. For reactions containing nsP2 protease, 4 μg of the purified protein was added to the cell lysate prior to the addition of any compound. Volumes of each reaction component is shown below:

**Table:** 

Condition	HEK293 lysate (3.45 mg/mL)	Reaction buffer^a^	nsP2 protease (1 mg/mL)	VSC (240 μM in DMSO)	VS or CA (250 μM in DMSO)
Lysate + **VSC**	40 μL	6 μL	0 μL	2 μL	2 μL
Lysate	40 μL	8 μL	0 μL	0 μL	2 μL
Lysate + nsP2 + **VSC**	40 μL	2 μL	4 μL	2 μL	2 μL
Lysate + nsP2	40 μL	4 μL	4 μL	0 μL	2 μL

a 25 mM HEPES pH 7.3, 1 mM DTT in H_2_O, 1% DMSO.

### 3.11 HEK293 Cell Lysate Pull-Down.

For biotin-streptavidin pull-down experiments, 2 μL of a 300 μM DMSO stock solution of clickable vinyl sulfone **VS** was added to 50 μL of HEK293 cell lysate (~170 μg total protein) and the mixtures were incubated at 24 °C for 30 min. Equal volumes of THPTA (6 mM stock in H_2_O) and CuSO_4_.H_2_O (12 mM stock in H_2_O) were premixed to generate the ligand-copper solution. For the click reaction, freshly prepared solutions of 10 μL biotin-PEG3-azide (60 μM stock in buffer), 20 μL ligand-copper premix, and 10 μL sodium ascorbate (12 mM stock in H_2_O) were added successively to cell lysates, and the mixture was incubated in the dark at 24 °C for 1 h. On completion, the reaction mixture was added to 30 μL of pre-equilibrated (3 washes with reaction buffer) streptavidin magnetic beads (NEB, cat. S1420S) and incubated at 24 °C for 15 min. The supernatant was removed, and the beads washed with 100 μL of the following, successively: 3 × 1% SDS in H_2_O, 1 × reaction buffer, 3 × 8 M urea in H_2_O, 1 × reaction buffer, 3 × 20% acetonitrile in H_2_O, 1 × reaction buffer. 40 μL of SDS-PAGE sample buffer [1:5 volumes of 10X reducing agent (NuPAGE; catalog number NP0009) to 2X SDS sample buffer (Novex, cat. LC2676)] was added to the beads and the mixture heated at 85 °C for 5 min. Samples were separated in a 4–12% Tris-Glycine SDS-PAGE (Novex, catalog number XP04125BOX). For control experiments cell lysate diluted in reaction buffer was pre-incubated with 2 μL of **3** (final concentration 1 μM) at 24 °C for 30 min. 2 μL of **VS** (final concentration 1 μM) was then added and the mixture incubated at 24 °C for another 30 min. Click reaction was carried out using the procedure described above. A control sample consisting of 50 μL cell lysate, 10 μL buffer and 40 μL H_2_O was incubated with 30 μL of pre-equilibrated magnetic streptavidin beads at 24 °C for 15 min. The beads were washed and prepared following the procedure described above.

For mass spectrometry (MS) analysis, SDS-PAGE gel lanes were excised and cut into 1 mm cubes and destained overnight. The samples were reduced with 5 mM DTT and alkylated with 15 mM iodoacetamide, then digested with 0.5 μg/μL of trypsin overnight at 37 °C. Peptides were extracted from the gel and cleaned using ZipTip pipette tips. The samples were analyzed as duplicate injections by LC-MS/MS using an Easy nLC 1200 coupled to a QExactive HF mass spectrometer (Thermo Scientific). Samples were injected onto an Easy Spray PepMap C18 column (75 μm id × 25 cm, 2 μm particle size) (Thermo Scientific) and separated over a 45 min gradient. The gradient for separation consisted of 5–38% mobile phase B at a 250 nL/min flow rate, where mobile phase A was 0.1% formic acid in water and mobile phase B consisted of 0.1% formic acid in 80% ACN. The QExactive HF was operated in data-dependent mode where the 15 most intense precursors were selected for subsequent fragmentation. Resolution for the precursor scan (*m/z* 350–1600) was set to 60,000 with a target value of 3 × 10^6^ ions. MS/MS scans resolution was set to 15,000 with a target value of 1 × 10^5^ ions, 100 ms injection time. The normalized collision energy was set to 27% for HCD. Dynamic exclusion was set to 30 s, peptide match was set to preferred, and precursors with unknown charge or a charge state of 1 and ≥ 8 were excluded. Raw data were processed using Proteome Discoverer (Thermo Scientific, version 3.1). Raw data were searched against a reviewed Uniprot human database (containing 20,355 sequences; downloaded January 2024), appended with a common contaminants database (MaxQuant, 245 sequences), and the CHIKV-nsP2 sequence. Enzyme specificity was set to trypsin, up to two missed cleavage sites were allowed; carbamidomethylation of Cys was set a static modification, oxidation of Met was set as a variable modification. A precursor mass tolerance of 10 ppm and fragment mass tolerance of 0.02 Da were used. Label-free quantification (LFQ) was enabled. Data were filtered based on a 1%/5% peptide/protein FDR, a minimum of 2 peptides, and quantitation in min. 2 samples was required for further analysis. LFQ abundances were Log_2_ transformed and missing values were imputed from a normal distribution with width 0.3 and downshift of 1.8. Statistical analysis was performed in Perseus (version 1.6.14.0) using the imputed values. Student’s t-tests (unpaired) were performed for the inhibitor-treated to control comparisons, and a p-value < 0.05 was considered statistically significant. LFQ log_2_ fold change ratios were calculated, and an absolute Log_2_ ratio ±−1 was considered significant.

## Supplementary Material

Supplement 1

## Figures and Tables

**Table 1:** Summary of the nsP2pro chemical probe (**3**) and negative control analog (**4**)

	Chemical Probe RA-0003022 (3)	Negative Control RA-0025453 (4)
Biological profile:		
Target Protein	CHIKV nsP2pro	CHIKV nsP2pro
In Vitro Potency (IC_50_)	40 nM	> 30 μM
Target Structure-Activity	Yes	Yes
Target Engagement	C478	
Kinetics (*k*_nact_/*K*_i_)	6000 M^−1^s^−1^	
Target Selectivity	>30-fold selective over 23 human and viral cysteine proteases	
GSH Reactivity (t_1/2_)	70 min	
Proteomic Selectivity	TAMRA-labelling and biotin-streptavidin pull-down	
Cellular Activity (CHIKV-nLuc EC_50_)	50 nM	> 10 μM
Cellular toxicity	None at 10 μM	None at 10 μM
Chemical properties:		
MW	350	392
clogP	2.4	3.0
Ligand Efficiency	0.4	
Polar Surface Area (PSA)	100 Å^2^	90 Å^2^
PAINS alerts	None	None
Chemical Stability	Stable at room temp. (pH 3–12)	Stable at room temp. (pH 3–12)
Aqueous Solubility	20 μM	240 μM
Aggregation by DLS	No effect at 100 μM	No effect at 100 μM

## Data Availability

The original contributions presented in the study are included in the article/Supplementary Material, further inquiries can be directed to the corresponding author. Chemical probe **3** and negative control **4** are available by request from the SGC and will be made available through commercial vendors.

## Abbreviations

CHIKV: Chikungunya virus
DIPEA: *N*,*N*-diisopropylethylamine
MAYV: Mayaro virus
TBTU: *N*,*N*,*N*′,*N*′-tetramethyl-O-(benzotriazol-1-yl)uronium tetrafluoroborate
VEEV: Venezuelan equine encephalitis virus
THPTA: Tris-hydroxypropyltriazolylmethylamine
SDS-PAGE: Sodium Dodecyl Sulfate–Polyacrylamide Gel Electrophoresis
TAMRA: Tetramethylrhodamine
HEK: Human Embryonic Kidney
