# Supplementary material for: A Covalent Chemical Probe for Chikungunya nsP2 Cysteine Protease with Antialphaviral Activity and Proteome-wide Selectivity
Source: Res Sq. 2024 Nov 12:rs.3.rs-5363451. Preprint. [Version 1] doi: 10.21203/rs.3.rs-5363451/v1 (PMC11601817; doi:10.21203/rs.3.rs-5363451/v1)
Supplement: Supplement 1 [file NIHPPRS5363451v1-supplement-1.pdf]

Supplementary Files

This is a list of supplementary files associated with this preprint. Click to download.

- SIFinal.pdf
- FileS217303248612.xlsx
- FileS117303248611.xlsx
